# Use of Normothermic Perfusion Machines in Lung Transplantation: Consensus Statement of the Italian Society of Organ and Tissues Transplantation Group With DELPHI Method

**DOI:** 10.3389/ti.2025.14762

**Published:** 2025-09-23

**Authors:** M. Schiavon, D. Bennett, M. Boffini, C. Carillo, A. Dell’Amore, J. Fumagalli, L. Luzzi, T. Pettenuzzo, L. Rosso, J. Scappin, A. Ali, D. Gregori, L. Melan, M. Martinato, the Study Group for Thoracic Organs Transplantation, F. Antonacci

**Affiliations:** ^1^ Thoracic Surgery Unit, Department of Cardiac, Thoracic Vascular Sciences and Public Health, University Hospital of Padova, Padova, Italy; ^2^ Respiratory Diseases Unit, Department of Medical Sciences, University Hospital of Siena, Siena, Italy; ^3^ Cardiac Surgery Division, Surgical Sciences Department, AOU Città della Salute e della Scienza di Torino, University of Turin, Turin, Italy; ^4^ Department of Thoracic Surgery and Lung Transplantation, University of Rome Sapienza, Policlinico Umberto I, Rome, Italy; ^5^ Department of Anaesthesia, Critical Care and Emergency, Fondazione Istituto di Ricovero e cura a Carattere Scientifico Ca’ Granda Ospedale Maggiore Policlinico, Milan, Italy; ^6^ Thoracic Surgery and Lung Transplant Unit, Department of Medical, Surgical and Neurosciences, University of Siena, Siena, Italy; ^7^ Institute of Anaesthesia and Intensive Care, University Hospital of Padua, Padua, Italy; ^8^ Thoracic surgery and Lung Transplantation Unit, Fondazione IRCCS Ca' Granda, Ospedale Maggiore Policlinico, Milano, Italy; ^9^ Statistic Unit, Department of Cardiac, Thoracic, Vascular Sciences and Public Health, University of Padova, Padova, Italy

**Keywords:** EVLP, consensus paper, lung transplantation, methodology, Delphi

## Abstract

**Background:**

*Ex vivo* lung perfusion (EVLP) is a technique for graft preservation, evaluation and treatment, that could expand donor pool for transplantation. Nevertheless, the wide spectrum of available platforms has generated disparities in use, outcome, and costs. This study is an attempt to create a national consensus on EVLP use by a group of experts from the Italian Society of Organ Transplantation.

**Methods:**

The 9-member promoting committee was divided into 3 groups to propose statements. Using the DELPHI method 27 experts (three from each of the 9 lung transplant centres) voted agreement to each statement in 3 rounds. The cutoff for acceptance was set at 80% agreement.

**Results:**

In the first vote, 52 statements were proposed, and an agreement was reached for 20 of them (38%). After revision, the second round resulted in a quorum for 36 out of 40 statements proposed (90%). At the third vote, agreement was confirmed for 36 statements (8 indications for use, 19 modalities for use, 13 evaluation parameters).

**Conclusion:**

The statements outlined in this document do not represent absolute guidelines, but rather recommendations. The statements selected and presented are therefore aimed to assist Italian clinicians in the use of an *ex vivo* normothermic perfusion platform in the right context.

## Introduction

Lung transplantation (LTx) is the preferred treatment option for patients with end-stage lung disease that has become unresponsive to medical therapy [1]. However, this treatment is still limited by the scarcity of suitable grafts (approximately 15%–30% of donors), which results in a significant mortality rate on the waiting list, estimated to be between 8%–13% [2]. In recent years, several strategies have been implemented to increase the donor pool. These include the use of lungs from extended-criteria donors [3] and DCDs [4].

The increased utilisation of non-standard grafts has been facilitated by the integration of *ex vivo* lung perfusion (EVLP) into clinical practice [5]. This procedure offers a potentially useful time window for both graft preservation and the evaluation and, possibly, reconditioning of lungs with questionable function [6, 7].

However, a range of protocols and devices are available for EVLP performance, including Lung Assist™ by Organ Assist^®^, XVIVO Perfusion System (XPS)™ by XVIVO^®^, Vivoline LS1™ by Vivoline Medical^®^, OCS™ by TransMedics^®^ and the TorEX Lung Perfusion System by Traferox^®^. The clinical potential of these machines is still under investigation [8]. This characteristic determines a wide heterogeneity of EVLP use in clinical practice between different centres [9], making comparison impossible.

The absence of recommendations or guidelines that can be utilised at a national level engenders challenges in the realm of reimbursement for device utilisation. Presently, the financial burden of these devices falls exclusively upon the budget allocated by transplant centres. The objective of this study is to deliberate and achieve a consensus on the utilisation of EVLP in Italy, with the aim of producing evidence-based recommendations to standardise clinical practice and minimise the cost-benefit ratio.

## Methods

**FIGURE 1:**
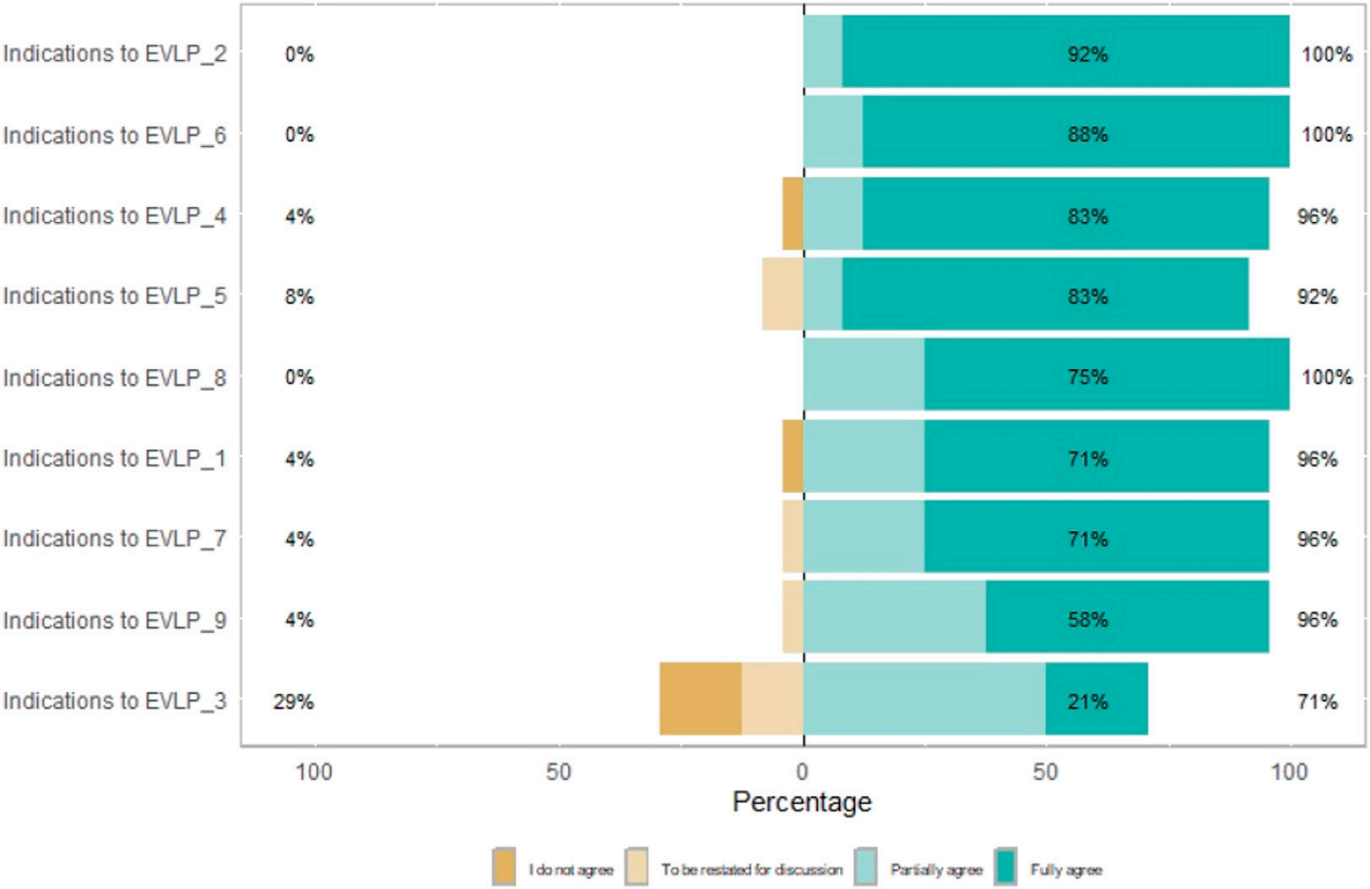
Representative map of the 9 lung transplant centres in Italy. The size of the blue circle is proportional to the number of transplants performed in the year 2023.

The present study was initiated by a working group of the Italian Society of Organ and Tissue Transplantation (SITO) with a view to developing a national consensus on the use of EVLP platforms. The Delphi method was employed to gather expert opinions and structure the recommendations, a technique that has gained wide recognition for its systematic approach to achieving consensus among diverse expert groups [10, 11]. The Delphi standard methodology and the limited availability of comparative randomised controlled trials (RCTs) precluded the application of a formal evidence grading system. A promoting committee was established, comprising nine experts selected according to criteria described in Table 1 from various disciplines, including five thoracic surgeons, two anaesthesiologists, one cardiac surgeon and one pulmonologist. These individuals represent five Italian lung transplant centres: Milan, Padua, Palermo, Siena and Turin. The committee was divided into three subgroups of three members each, tasked with drafting statements in three main categories: indications for use, operational methods, and evaluation parameters. Directors from the nine Italian lung transplant centres (see Figure 1; Table 2) nominated 27 experts (thoracic surgeons, anaesthesiologists and pulmonologists) to participate in the consensus process.

**TABLE 1:** Selection criteria for expert committee.

Selection criteria
Clinical activity more than 5 years
Participation in at least 10 EVLP procedures
At least 5 publications in the field of lung transplantation

**TABLE 2:** EVLP activity for each centre.

Transplant centre	Year of EVLP activity beginning	Volume activity
Bergamo	2017	15
Bologna	2019	9
Milano	2011	71
Padova	2011	62
Palermo	2015	2
Pavia	2017	4
Roma	2012	6
Siena	2016	16
Torino	2011	49

The proposed statements were evaluated using a four-point Likert scale in the first two rounds, followed by a dichotomous response (agreement/disagreement) in the third round. Furthermore, participants were granted the opportunity to provide commentary and substantiate their selections. The Delphi method’s structured feedback cycles are particularly well-suited to areas with limited empirical evidence, such as evolving practices in EVLP [12, 13]. The data were collected and managed via a survey developed in the REDCap (Research Electronic Data Capture) platform, which is hosted at the Unit of Biostatistics, Epidemiology, and Public Health, Department of Cardiac-Thoracic-Vascular Sciences and Public Health at the University of Padua [14, 15]. The Unit of Biostatistics, Epidemiology, and Public Health provided comprehensive support for the entire data collection and analysis process. REDCap is a secure, web-based software platform designed to support data capture for research studies. It provides an intuitive interface for validated data capture, audit trails for tracking data manipulation and export procedures, automated export procedures for seamless data downloads, and procedures for data integration and interoperability with external sources. A consensus threshold of 80% was established for the acceptance of the statement. Statements that did not meet the required standard were subjected to a process of refinement, informed by in-depth discussions and a review of the relevant literature. This iterative process was undertaken to ensure scientific rigour and alignment with best practices in consensus methodologies [16, 17].

**FIGURE 2:**
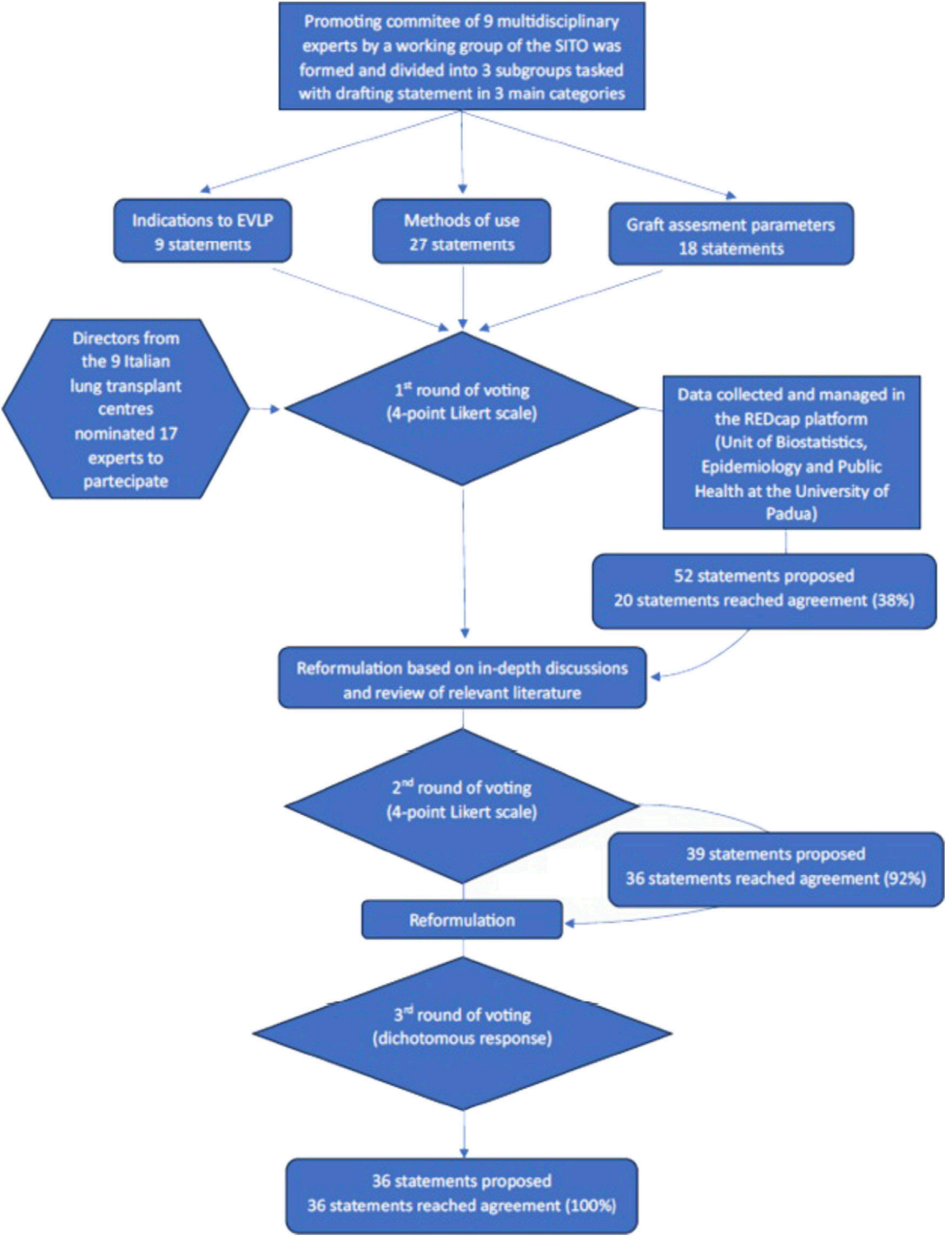
Flowchart of the statement development and voting process.

In order to avoid the introduction of bias, responses were collected anonymously. Furthermore, participant demographics (e.g., educational background and workplace) were processed exclusively in aggregate form, with the purpose of describing the panel of experts. Prior to commencing the survey, the participants were provided with a comprehensive overview of the data processing procedures. Statistical analyses, performed using R software, calculated agreement percentages and assessed response consistency. These tools are frequently employed in health research to validate consensus processes and quantify agreement [18]. However, it is important to acknowledge the limitations of the Delphi method. Firstly, there is the issue of expert bias, which arises from the selection of experts. This selection may influence the generalisability of the findings. Secondly, there is the lack of external validation, which arises from the method’s reliance on shared expert opinions and knowledge without direct experimental verification. Notwithstanding these limitations, the Delphi method continues to be recognised as a established approach for generating expert-driven recommendations in fields with limited robust evidence [10, 19]. A flowchart illustrating the activities undertaken can be found in Figure 2.

## Results

All 27 experts took part in the three votes. Following extensive deliberations, a consensus was reached on a total of 36 statements, encompassing 8 indications for use, 16 on methods of use, and 12 on graft assessment. A total of 52 statements were proposed during the first vote (see Supplementary Tables S1–S3), and agreement was reached for 20 of them (38%). It is evident from Supplementary Figures S1–S3 that none of the proposed statements achieved a disagreement rate of more than 80% among the voting experts. The 32 statements that did not reach the required agreement were then reformulated by the respective committees (see Supplementary Tables S4–S6). In the subsequent vote, 40 statements were submitted with supporting literature: 8 on indications for use, 19 on methods of use and 13 on graft assessment parameters. The results of the vote established a quorum for a total of 36 statements, with four statements failing to reach the requisite level of agreement (see Supplementary Figures S4–S6). In the most recent dichotomous vote (see Supplementary Figures S7–S9), consensus was reached for a total of 36 statements (see Tables 3–5).

**TABLE 3:** Indications to EVLP.

Statement	Consensus
EVLP can be used as an effective technique for organ preservation	88.9%
EVLP is a useful platform for organ assessment	100.0%
There is currently no strong clinical evidence for a role of EVLP in active organ reconditioning	92.6%
The use of EVLP may find indication in both DBD and DCD donation of any class	100.0%
EVLP can be used for graft, regardless of the clinical condition of the recipient	96.3%
The use of EVLP is proposed in cases of donors with questionable organ function, or not evaluable at harvest	100.0%
EVLP is usable in the case of logistical or clinical issues that have the potential to increase ischemia time	100.0%
EVLP is not recommended for use in case of irreversible structural damage of the graft	100.0%

**TABLE 4:** Methods of use.

Statement	Consensus
There are three most widely used of EVLP in clinical practice (Lund, Toronto, OCS), but no evidence exists, at present, regarding the superiority of one over the others	100.0%
There are, at present, no differences in clinical results obtained between perfusions with acellular and cellular solution with concentrated hematins	100.0%
Achievement of target flow must occur in a congruent time concomitant with lung rewarming	100.0%
It is recommended to maintain pulmonary arterial pressures less than 15–20mmHg to reduce the risk of developing pulmonary oedema	100.0%
In case of lung split during machine reperfusion, as well as in monopulmonary reperfusion, adjustment of target flow to the monopulmonary condition is mandated, maintaining control of mean PAP and pulmonary resistances as much as possible	100.0%
It is recommended that lung ventilation should not begin until temperatures between 32 °C–34 °C have been reached	100.0%
During the reperfusion process, it is recommended to maintain a respiratory rate of 7–12 acts/minute, and in any case always less than 20 acts/minute	100.0%
Regarding static EVLP, it is recommended to assess lung function after a recruitment manoeuvre having the purpose of reopening collapsed lung regions	100.0%
When performed for the purpose of portable EVLP (early normothermic perfusion), it is not strictly necessary to keep the lungs inflated at the end of retrieval, as hypothermic transport prior to graft insertion in the machine is not provided	96.3%
When performed during EVLP, there is no evidence of superiority of one mode of recruitment over another	100.0%
Pronation of the lungs during EVLP can be considered	100.0%
In case of “minor” air leakage from the lung parenchyma that does not complicate parenchymal recruitment and organ evaluation, attempted breach repair with sutures or staplers is not recommended	96.3%
In case of lung parenchyma deflation or failure to achieve adequate recruitment in the absence of problems with the ventilatory system, having verified proper circuit closure and the absence of frank areas of parenchymal air leakage, flexible bronchoscopy through the dedicated operative canal is recommended to check for secretions and aspirate them	100.0%
In the case of lung split during machine reperfusion, or in the case of monopulmonary reperfusion, reduction of tidal volume from defined criteria for bipulmonary reperfusion is critical	100.0%
Once organ suitability is defined, there is, at present, no evidence of the best timing and mode of lung separation and preservation of the second lung (hypothermia vs. EVLP continuation)	100.0%
Since there is, at present, no evidence to support a better outcome with the use of one class of antimicrobials than the others, the decision on the use of the type and dosage of antimicrobials during EVLP is deferred to the experience of the transplant centre	100.0%

**TABLE 5:** Graft assessment parameters in EVLP.

Statement	Consensus
Evaluation of graft quality during EVLP is based on multiple standard physiological and objective parameters. One parameter alone is not sufficient to assess graft quality. In addition, at least two endobronchial assessments during *ex vivo* reperfusion phases are desirable	100.0%
For all evaluation parameters, the trend over time should be considered more relevant than the absolute value (best or worst)	100.0%
Flexible bronchoscopy through dedicated Bronco-Port is recommended to assess the presence of foamy fluid (oedema), haemorrhagic fluid, repletion with purulent secretions, or signs of aspiration	100.0%
It is not recommended to use grafts in which it is verified through bronchoscopy during EVLP of frank plasmorrhea and signs of aspiration, or repletion of purulent secretions	100.0%
Visual inspection at the end of lung parenchyma recruitment is recommended to detect features such as haemorrhagic infarction, appearance of infarct areas, and other abnormalities that may affect lung function and its suitability for transplantation	100.0%
Palpatory inspection of the graft at the end of lung parenchyma recruitment is recommended to detect features such as decreased elasticity of the parenchyma itself or increased weight of the various areas, appearance of areas of thickening, and other abnormalities that may affect lung function and its suitability for transplantation	96.3%
The assessment of PaO2/FiO2 value, in isolation, is never sufficient for the definitive evaluation of the goodness of the graft	100.0%
At the end of adequate recruitment period and performance of hemogasanalysis in EVLP, PaO2/FiO2 values 350mmHg (or 300mmHg in case of using cellular solution) indicate doubtful graft performance. Notwithstanding, we defer to the experience of the transplant centre to evaluate graft quality according to all the multiple physiological and objective parameters necessary for this evaluation	100.0%
Evaluation of pulmonary vascular resistance trends during the procedure is recommended. An increase in resistances should cause organ damage to be considered	100.0%
Continued evaluation of perfusate leakage in the bell is recommended. Once anastomotic defects or frank parenchymal injury have been excluded, evolution to pulmonary oedema should be considered. Where feasible, assessment of weight change during the procedure may be indicative of possible organ oedema	100.0%
Evaluation of static compliance of the isolated organ is recommended. Values of less than 70mL/cmH2O at the end of the evaluation, or worsening over time, should be considered doubtful graft performance	96.3%
Radiography is recommended, if possible, to better define any regionality of organ damage (signs of oedema, imbibition, interstitial overload, parenchymal lesions); radiography alone cannot preclude organ use, only guide the decision	100.0%

## Discussion

Normothermic perfusion platforms are assuming an increasing role in lung transplantation as they represent an option for graft preservation, evaluation and possible reconditioning [20]. However, the broad spectrum of indications and protocols can prove perplexing and give rise to considerable divergence within the domain of evaluation modalities, outcomes and management costs. This is of particular importance in Italy, where a reimbursement procedure for the use of the device has not yet been implemented and the lack of shared recommendations limits the legislator. Consequently, the pre-eminent authorities in the domain of lung transplantation within the nation have determined the imperative to embark upon the formulation of a consensus on the utilisation of this apparatus, encompassing its indications, methodologies of application, and evaluation criteria for lungs subjected to EVLP.

The authors elected to prioritise percentage consensus rates over hierarchical levels of evidence in their methodological approach. Please refer to the supplementary materials for a comprehensive overview of the consensus results and to the following references for a detailed mapping of the sources.

### Indications to EVLP

Experts have agreed that, according to the available literature, EVLP has shown to allow extending the preservation window, ranging from few hours (4–6 h) [21–23], to extended durations (exceeding 12 h) [24]; Reported clinical experiences show a rate of graft unsuitability after EVLP <25% and comparable post-transplant outcome in EVLP treated graft recipients [25].

EVLP is also indicated in lung graft evaluation [7, 20, 26, 27], both from DBD [28] and DCD donors [27, 29–34]. In particular, the utilisation of EVLP should be contemplated in instances of doubtful or non-assessable organ function at retrieval [30, 35, 36]. In this context, EVLP platforms have also been shown to be effective in highlighting graft issues (infections, inflammation) that are not apparent in initial donor evaluation [36–38]. Furthermore, their utilisation should be contemplated in instances where logistical or clinical concerns have the capacity to prolong ischemic times [39], thereby facilitating a comprehensive evaluation and optimising the suitability of grafts for transplantation, even across substantial geographical distances [40, 41].

It was determined by the collective opinion of the experts that the decision to utilise EVLP should be made irrespective of the condition of the recipient, given that the utilisation of EVLP has already been documented for both standard [41] and urgent recipients [42]. In the absence of exclusion criteria for the utilisation of EVLP for recipients [43], as outlined in referral guidelines [44, 45], lungs from donors exhibiting significant infection, such as full-blown pneumonia, purulent discharge or overt signs of aspiration during bronchoscopy, and severe irreversible structural damage to the graft, should be excluded from *ex vivo* perfusion [6, 43].

Finally, despite the plethora of reported successes in the literature [30, 35, 37, 46], experts concur that the role of EVLP in active lung graft reconditioning remains unrecognised, largely due to conflicting results [23, 26, 47]. The necessity for prospective multicentre randomised studies is evident in order to achieve a more precise definition of this issue.

### Methods of Use

It has been posited by experts in the field that there are three primary EVLP protocols (Lund, Toronto and OCS) [6, 9, 20, 48], though at present, there is an absence of studies that directly compare the relative merits of these protocols. The impact of the individual parameters of each device and protocol on organ function after EVLP, PGD development and post-operative outcome has yet to be evaluated. The optimal approach remains to be determined, as the debate surrounding the superiority of early versus delayed normothermic perfusion persists [49, 50]. The ambiguity arises from the ongoing discourse surrounding the optimal atrium configuration, namely, whether to employ an open or closed approach [21, 51–53]. The prevailing consensus is that the decision regarding the selection of the EVLP system to be employed rests with the individual transplant centre, contingent upon its preferences, experience, and accessibility. Furthermore, at this time, the results of studies comparing different perfusion solutions (cellular vs. acellular) remain inconclusive [54–56]. For short perfusion times, perfusion solutions with the addition of blood might offer an advantage for lung assessment [57]. Conversely, in prolonged EVLP, the use of acellular solutions might be advantageous in order to avoid the harmful effects of haemolysis [58].

A consensus was achieved on the modalities of circulation and ventilation, with particular reference to the timing of achieving the target flow [22, 28, 59–66] and the initiation of ventilation [6, 20, 67], as illustrated in Table 4. Specifically, a standardised protocol should be established to concurrently increase pulmonary blood flow and graft core temperature at the initiation of EVLP, in accordance with the target flow rate intended for maintenance during the procedure. Maintaining pulmonary arterial pressures below 15–20 mmHg was also recommended in order to avoid the development of oedema [53, 58, 66, 68, 69]. Furthermore, it was advised that low tidal volume (below 8 mL/kg predicted body weight) and a respiratory rate always below 20 acts/minute should be maintained to avoid ventilator-induced lung injury [6, 70–77]. Two other statements make specific recommendations for portable or static systems: In the context of portable EVLP (early normothermic perfusion), experts do not perceive a requirement to maintain lung inflation at the conclusion of retrieval as is customary [24]. This is due to the fact that hypothermic transport prior to graft insertion in the machine is not anticipated [60, 68], thereby circumventing the risk of barotrauma injury [78]. Conversely, for static EVLP, it is advised to execute recruitment manoeuvres prior to graft function evaluation in order to ensure the homogenisation of ventilation distribution [6, 79, 80]. However, it has been specified that there is an absence of evidence to suggest that one recruitment modality is superior to another. In instances where air leakage from the parenchyma does not complicate recruitment, the repair of breaches with sutures or staplers is generally discouraged. This is due to the experience accumulated by experts over the years, which has shown that such procedures can cause lung damage, which in turn can exacerbate the progression of EVLP.

In the event of lung deflation, once potential causes associated with the circuit itself have been excluded, it is advised to undertake a flexible bronchoscopy to ascertain the presence of secretions and to aspirate them, if necessary. In the absence of other causative factors, the occurrence of deflation is a salient factor in the potential for graft injury.

In order to further improve the procedure, experts recommend considering the pronation of the lungs in EVLP, if safely possible; in fact, this could improve graft function, avoiding the development of oedema in the declivous regions [81, 82]. A wide range of options is available with respect to the type and dosage of antimicrobials, as no superior treatment has been identified [36, 37, 83–88].

It is evident that, upon ascertaining the suitability of organs, the optimal temporal parameters for lung separation remain to be elucidated. Indeed, some platforms allow the perfusion of one lung to continue during the implantation of the contralateral lung [89], further reducing cold ischemia periods. However, the potential benefit of this procedure [90] has yet to be demonstrated by comparative studies, and experts have agreed that further investigation is required. In any case, experts concur that, in the event of a split during EVLP or mono-pulmonary perfusion, adjustment of ventilation and circulation parameters is imperative [91, 92].

### Graft Assessment Parameters in EVLP

The expert emphasised that graft assessment during EVLP is based on multiple parameters, since one parameter alone is not sufficient to guarantee graft suitability for transplantation. Moreover, it is imperative to acknowledge that the trend over time holds greater significance than the absolute value (best or worst) in relation to all evaluation parameters. All available protocols [60, 93–96] recommend that the decision regarding implantation should be made subsequent to consideration of the stability of lung perfusion and ventilation parameters, the PaO2/FiO2 ratio, and finally the organ condition based on visual and tactile examination.

With regard to the PaO2/FiO2 value, it is widely accepted among experts that a single sample is never sufficient for the definitive assessment of graft quality. Furthermore, a PaO2/FiO2 ratio of less than 350 mmHg (or less than 300 mmHg when using a cellular solution) should raise suspicion of poor graft performance. It has been posited that PaO2 does not always reflect the condition of the lung graft [97] and that the PaO2/FiO2 confidence interval for acceptance can vary greatly depending on the type of solution used (cellular or acellular) [98], due to the linearization of the relationship between oxygen content and PaO2 that occurs with acellular perfusate.

It has been demonstrated that a repeated objective examination during EVLP may result in the identification of areas of the lung parenchyma that are more susceptible to the accumulation of hydrostatic fluid, which can potentially lead to the development of pulmonary oedema [99]. The rate of fluid consumption in the reservoir, and, where feasible, the assessment of weight change along the procedure, should be considered a marker of organ oedema development [100].

Conversely, experts have recommended considering a questionable graft performance in cases of increased vascular resistance, static compliance with values below 70mL/cmH2O at the conclusion of the evaluation, or a deterioration of these parameters over time [80]. The findings of numerous studies [101, 102] demonstrate that these parameters serve as effective quantitative indicators of lung function, providing a valuable addition to the existing body of research.

Instrumental examinations have been considered equally fundamental: flexible bronchoscopy is useful for assessing the presence of bronchorrhea and signs of aspiration, or the repletion of purulent secretions that contraindicate the use of the graft for transplantation [95, 103, 104] and at least two endobronchial assessments during *ex vivo* reperfusion would be desirable. With regard to X-ray examinations, a special compartment for safely performing X-rays is provided in static platforms [95], but feasibility has also been described for portable platforms [105]. Finally, it must be acknowledged that, in contrast to conventional chest radiographs whose usefulness has been called into question [106–109], EVLP radiographs offer isolated images of the donor lungs with enhanced contrast. This allows for radiographic findings in EVLP that are associated with the outcome of lung transplantation [110, 111].

In light of the aforementioned factors, it is recommended by experts that an X-ray be performed in EVLP. However, it is also stressed by these experts that the imaging results should be considered as only one part of the evaluation. As previously stated, radiographs have the capacity to yield confounding data [107], and consequently, they must be evaluated and interpreted in conjunction with the extensive array of decision-making values provided by EVLP platforms.

## Conclusion

In conclusion, this consensus statement, which was reached by the Delphi method, represents a shared agreement among 27 experts from nine Italian lung transplant centres regarding normothermic *ex vivo* lung perfusion. In view of the paucity of multicentre randomised prospective studies comparing the three major EVLP protocols in use, it is imperative to emphasise that the statements outlined in this document do not represent absolute guidelines, but rather recommendations that are the direct expression of the experts' shared opinion and knowledge. The statements selected and presented are therefore aimed at assisting Italian clinicians in the complex decision to reject an organ, accept it for transplant after a period of cold ischemia, or use an *ex vivo* normothermic perfusion platform in the right context, with shared modalities and evaluation criteria. However, it is imperative for practitioners to acknowledge that, by their very nature, these statements cannot be regarded as definitive, as this is a newly introduced and evolving field with considerable potential and future prospects. Furthermore, the consensus presented in our manuscript reflects perspectives from a single national context. While the implementation of an external validation process to assess the transferability of the consensus across diverse healthcare systems and cultural contexts would undoubtedly enhance the robustness, generalizability and applicability of the findings, such an endeavour was beyond the scope and resources of the present project. Nevertheless, this may represent a significant direction for future research.

Notwithstanding the aforementioned limitations, this inaugural national document on the utilisation of *ex vivo* perfusion systems for lung transplantation has the potential to serve as a valuable clinical instrument. Moreover, it could serve as a unifying point for the pursuit of economic reimbursement for such procedures, a factor that presently imposes significant constraints on the dissemination of this pivotal technology within the transplant domain.

## Group members of Thoracic Organs Transplantation

F. Antonacci (Division of Thoracic Surgery, IRCCS Azienda Ospedaliero Universitaria Di Bologna, Bologna, Italy), A. Benigni (Chirurgia Generale 3 - Trapianti Addominali, Department of Surgery, ASST Papa Giovanni XXIII, Bergamo, Italy), M. Beretta (Chirurgia Generale 3 - Trapianti Addominali, Department of Surgery, ASST Papa Giovanni XXIII, Bergamo, Italy), A. Bertani (Division of Thoracic Surgery and Lung Transplantation, Chest Center, IRCCS ISMETT (Istituto Mediterraneo per i Trapianti e Terapie ad alta Specializzazione), Palermo, Italy), P. Bertoglio (Division of Thoracic Surgery, IRCCS Azienda Ospedaliero Universitaria Di Bologna, Bologna, Italy), C. Catelli (Lung Transplantation Unit, Department of Medical, Surgical and Neurosciences, University of Siena, Siena, Italy), A. Diso (Department of Thoracic Surgery and Lung Transplantation, University of Rome Sapienza, Policlinico Umberto I, Rome, Italy), F. Franchi (Lung Transplantation Unit, Department of Medical, Surgical and Neurosciences, University of Siena, Siena, Italy), D. Giunta (Division of Thoracic Surgery and Lung Transplantation, Chest Center, IRCCS ISMETT (Istituto Mediterraneo per i Trapianti e Terapie ad alta Specializzazione), Palermo, Italy), M. Marro (Cardiac Surgery Division, Surgical Sciences Department, AOU Città della Salute e della Scienza di Torino, University of Turin, Turin, Italy), G. Melloni (Division of Cardiac Surgery, IRCCS San Matteo Hospital, Pavia, Italy), M. Nosotti (Thoracic surgery and Lung Transplantation Unit, Fondazione IRCCS Ca' Granda, Ospedale Maggiore Policlinico, Milano, Italy), A. Pangoni (Division of Thoracic Surgery and Lung Transplantation, Chest Center, IRCCS ISMETT (Istituto Mediterraneo per i Trapianti e Terapie ad alta Specializzazione), Palermo, Italy), S. Pelenghi (Division of Cardiac Surgery, IRCCS San Matteo Hospital, Pavia, Italy), C. Pellegrini (Division of Cardiac Surgery, IRCCS San Matteo Hospital, Pavia, Italy), D. Pinelli (Chirurgia Generale 3 - Trapianti Addominali, Department of Surgery, ASST Papa Giovanni XXIII, Bergamo, Italy), F. Ruberto (Department of Thoracic Surgery and Lung Transplantation, University of Rome Sapienza, Policlinico Umberto I, Rome, Italy), N. Sella (Institute of Anaesthesia and Intensive Care, University Hospital of Padua, Padua, Italy), E. Simonato (Cardiac Surgery Division, Surgical Sciences Department, AOU Città della Salute e della Scienza di Torino, University of Turin, Turin, Italy), P. Solidoro (Cardiac Surgery Division, Surgical Sciences Department, AOU Città della Salute e della Scienza di Torino, University of Turin, Turin, Italy), P. Solli (Division of Thoracic Surgery, IRCCS Azienda Ospedaliero Universitaria Di Bologna, Bologna, Italy).

## Abbreviations

DBD, Donation after Brain Death; DCD, Donation after Circulatory Death; EVLP, *Ex vivo* lung perfusion; SITO, Italian Society of Organ and Tissues Transplantation; LTx, Lung transplantation; OCS, Organ Care System; REDCap, Research Electronic Data Capture platform.
