# Supplementary figures and images for: Use of Normothermic Perfusion Machines in Lung Transplantation: Consensus Statement of the Italian Society of Organ and Tissues Transplantation Group With DELPHI Method
Source: Transpl Int. 2025 Sep 23;38:14762. doi: 10.3389/ti.2025.14762 (PMC12500477; doi:10.3389/ti.2025.14762)

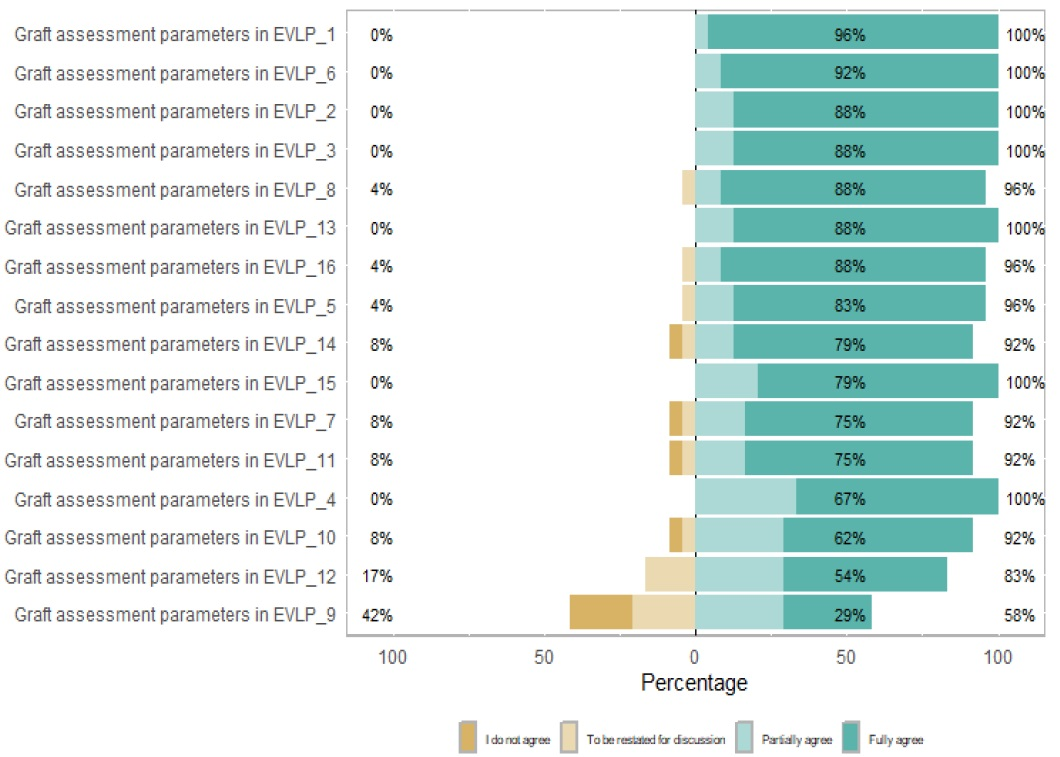

Supplement: Supplementary file 1 [file Image3.tiff]

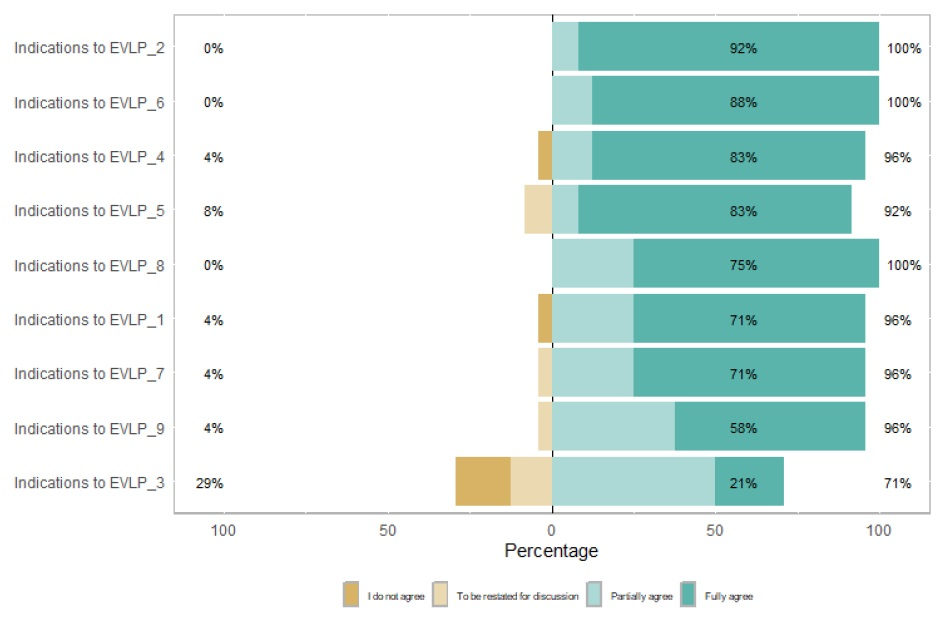

Supplement: Supplementary file 2 [file Image1.tiff]

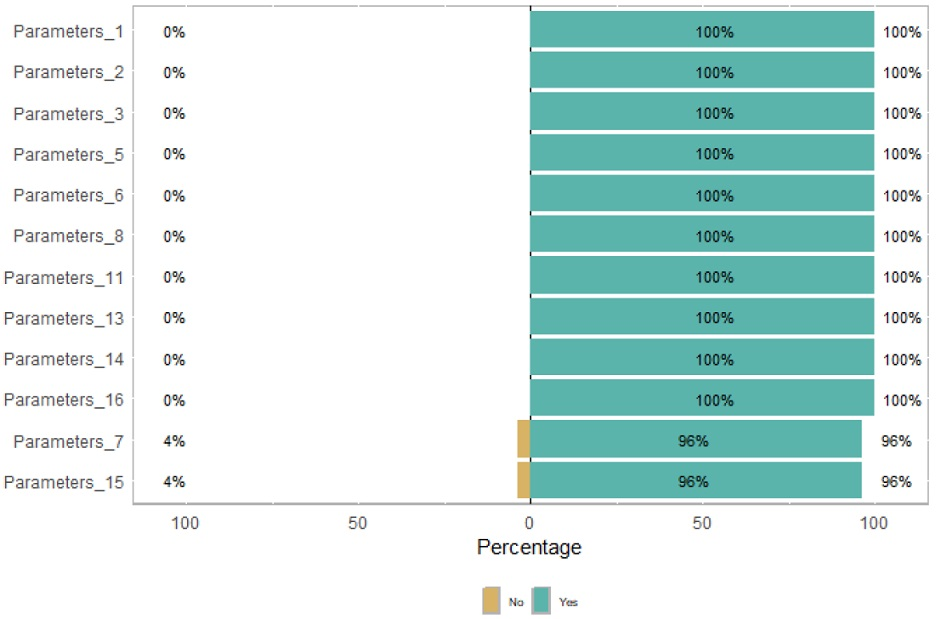

Supplement: Supplementary file 3 [file Image9.tiff]

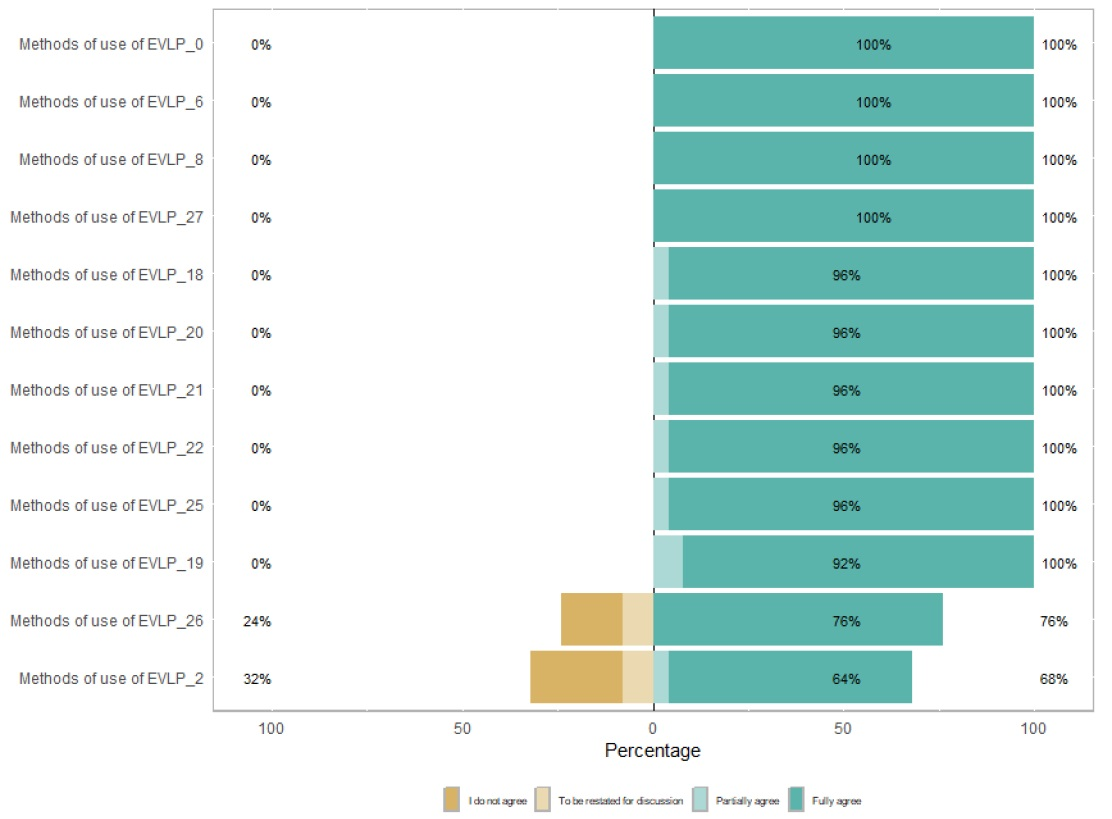

Supplement: Supplementary file 4 [file Image5.tiff]

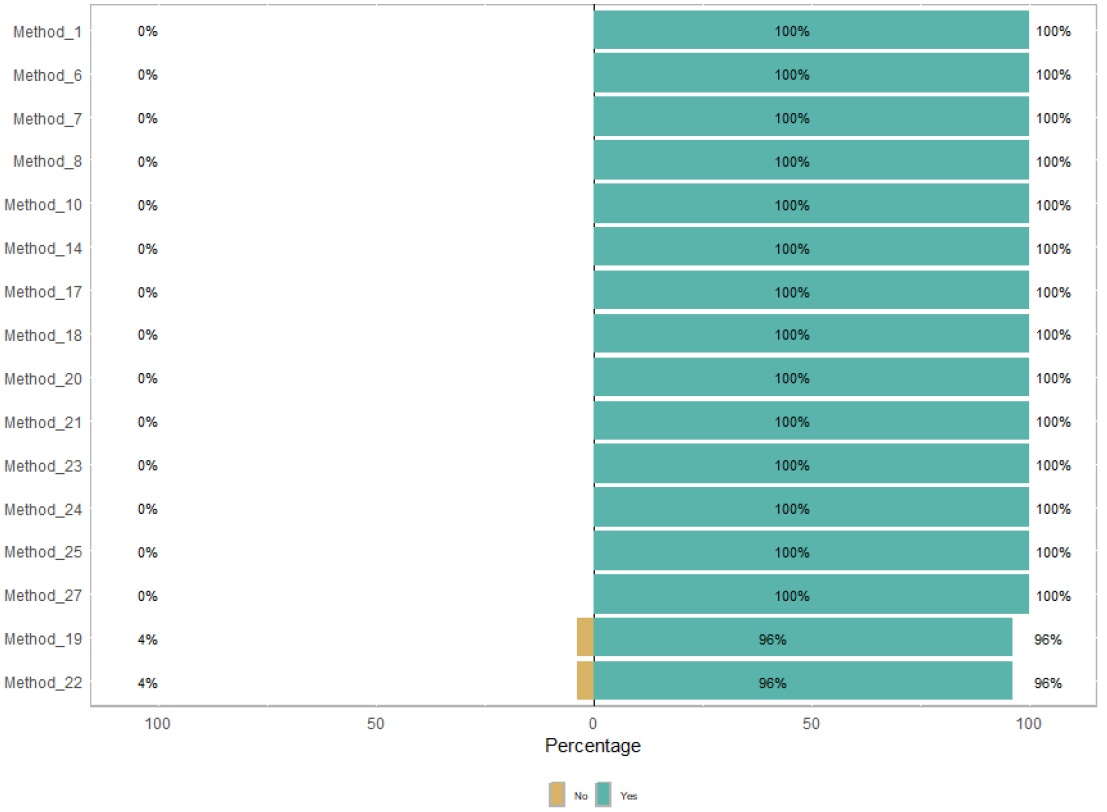

Supplement: Supplementary file 5 [file Image8.tiff]

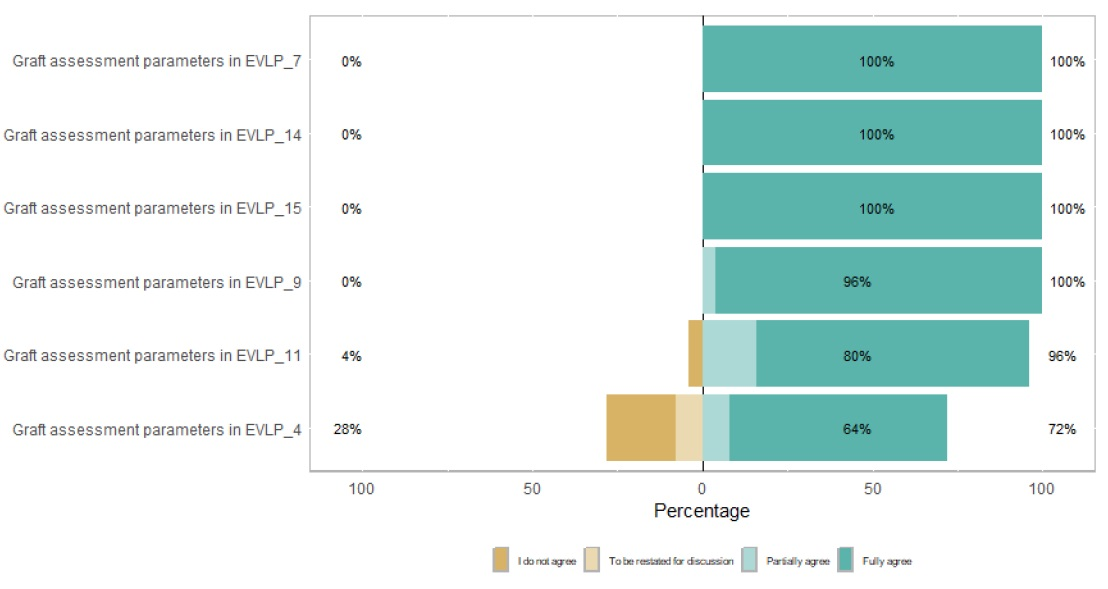

Supplement: Supplementary file 6 [file Image6.tiff]

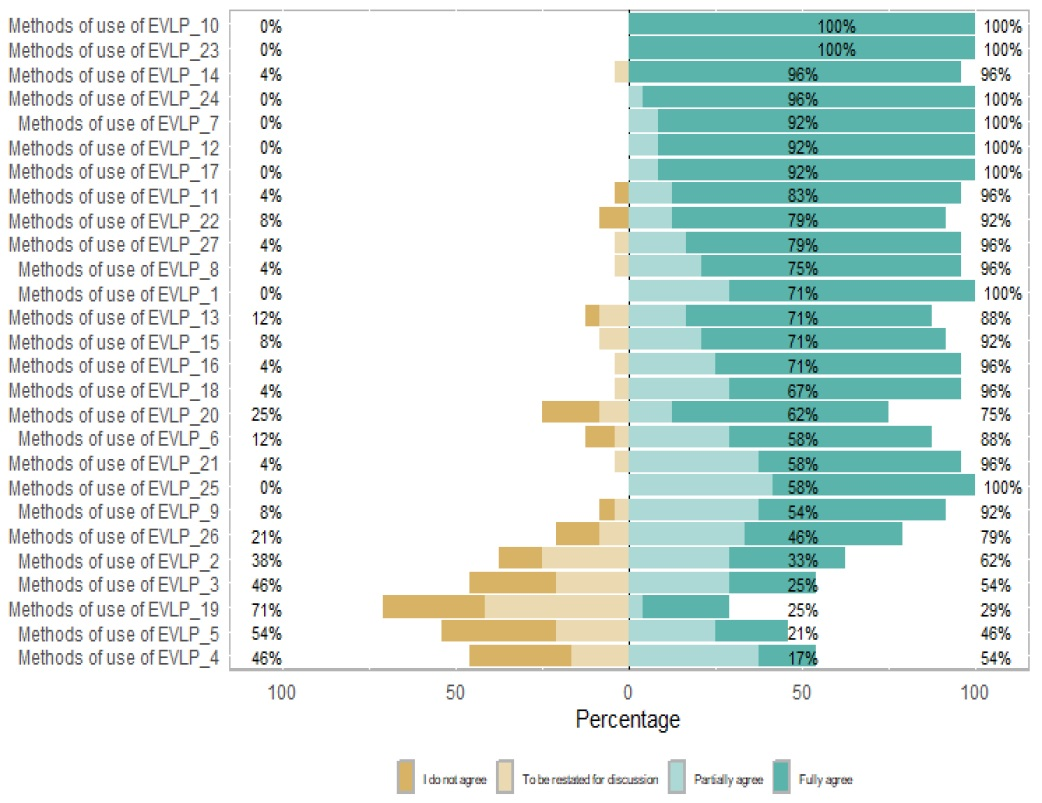

Supplement: Supplementary file 7 [file Image2.tiff]

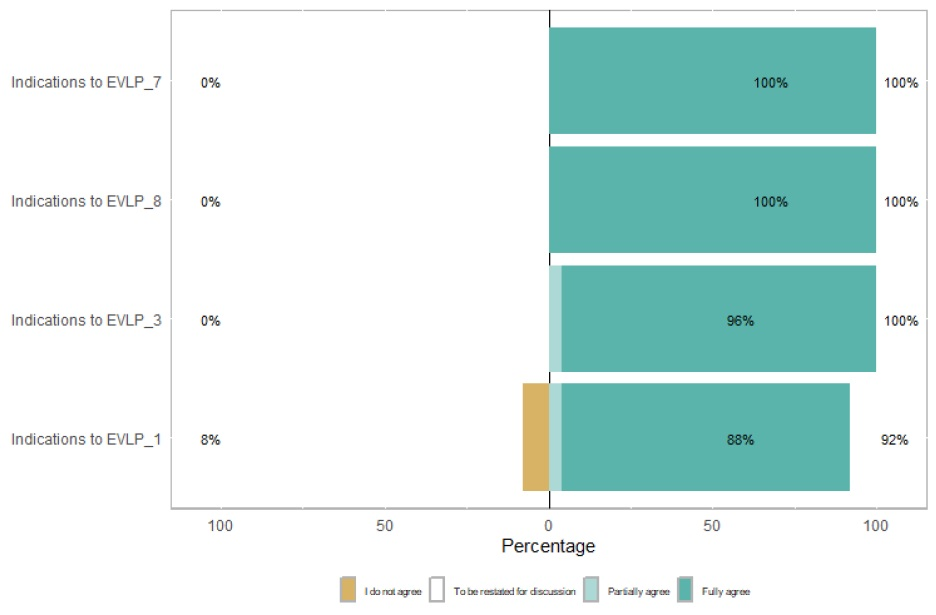

Supplement: Supplementary file 8 [file Image4.tiff]

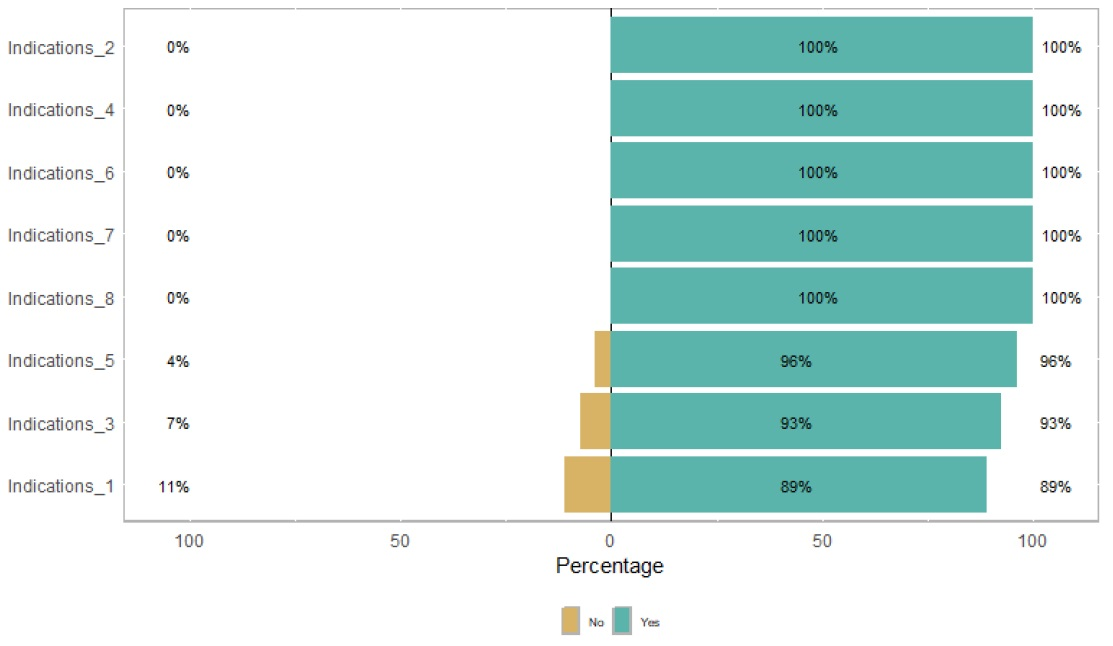

Supplement: Supplementary file 10 [file Image7.tiff]
