# Supplementary material for: Use of Normothermic Perfusion Machines in Lung Transplantation: Consensus Statement of the Italian Society of Organ and Tissues Transplantation Group With DELPHI Method
Source: Transpl Int. 2025 Sep 23;38:14762. doi: 10.3389/ti.2025.14762 (PMC12500477; doi:10.3389/ti.2025.14762)
Supplement: Supplementary file 9 [file DataSheet1.docx]

# **Use of normothermic perfusion machines in lung transplantation: consensus statement of the Italian Society of Organ and Tissues Transplantation group with DELPHI method**

**Supplementary materials**

**[the order in which supplementary materials should appear is: Table S1, Figure S1, Table S2, Figure S2, Table S3, Figure S3, Table S4, Figure S4, Table S5, Figure S5, Table S6, Figure S6, Figure S7, Figure S8, Figure S9]**

**Figure S1:** Results of the first vote related to Indications to EVLP.

**Figure S2:** Results of the first vote related to Methods of use.

**Figure S3:** Results of the first vote related to Graft assessment parameters.

**Figure S4:** Results of the second vote related to Indications to EVLP.

**Figure S5:** Results of the second vote related to Methods of use.

**Figure S6:** Results of the second vote related to Graft assessment parameters.

**Figure S7:** Results of the third vote related to Indications to EVLP.

**Figure S8:** Results of the third vote related to Methods of use.

**Figure S9:** Results of the third vote related to Graft assessment parameters.

**Table S1 - Initial statements – indications to EVLP**

| 1) The use of EVLP is an effective and safe technique as an organ preservation modality |
| --- |
| 2) EVLP is a useful platform for organ evaluation |
| 3) There is currently no strong evidence on the use of EVLP as an active graft reprocessing platform. |
| 5) EVLP can be used for graft, regardless of the clinical condition of the recipient. |
| 6) The use of EVLP is proposed in cases of donors with doubtful organ function, or not assessable at harvest. |
| 7) EVLP can be used in the case of logistical or clinical issues that could potentially increase ischaemia time. |
| 8) EVLP is not advisable in the case of signs of irreversible structural graft damage |
| 9) There are no specific indications for the exclusive use of static or portable EVLP at the present time. |

**Table S2 - Initial statements – methods of use**

| 1) As there is no evidence to support early or delayed normothermic perfusion, the decision of the type of EVLP system remains at the discretion of the centre, according to its preference and availability. |
| --- |
| 2) The use of human albumin in the perfusate is recommended to improve the hyper-oncotic power of the perfusion solution. |
| 3) The addition of dextran to the solution limits the need for replacement of the hyper-oncotic solution over time. |
| 4) Perfusion with solution supplemented with whole blood offers the best results in the preservation and homeostasis of the lung over time. |
| 5) Perfusion with solution supplemented with whole blood has no application in the clinical setting due to the difficulties in obtaining it. |
| 6) There are currently no differences in the clinical results obtained between perfusion with acellular solution and cellular solution with concentrated haematias. |
| 7) The achievement of the target flow must take place in a congruent time and concomitant with the heating of the lung. |
| 8) It is recommended to maintain systolic pulmonary arterial pressures below 20mmHg to reduce the risk of developing pulmonary oedema. |
| 9) There are currently no differences in lung preservation results between the different target flows used with the different device protocols. |
| 10) In the case of lung splitting during in-machine reperfusion, as well as in monopulmonary reperfusion, adaptation of the target flow to the monopulmonary condition is mandated, while maintaining control of mean PAP and lung resistance as much as possible. |
| 11) There is no difference in pulmonary preservation results between closed and open atrium systems. |
| 12) Once the preferred atrium system is chosen, it is mandated that the atrial chamber is fully open or closed throughout the duration of perfusion. |
| 13) The goal of ventilation during reperfusion is to maintain protective ventilation settings during functional evaluation of the organ. |
| 14) It is recommended that lung ventilation does not start until temperatures between 32-34°C have been reached. |
| 15) During the reperfusion process it is recommended to maintain low PEEP (5-7 cmH2O), even in the case of lungs that were initially de-recorded and visibly atelectatic at the time of donor retrieval. |
| 16) During the reperfusion process, the maintenance of low tidal volume (approximately 5-7 mL/kg predicted donor body weight) is recommended, even in the case of initially de-recorded and visibly atelectatic lungs at the time of donor collection. |
| 17) During the reperfusion process, it is recommended to maintain a respiratory rate of 7-12 acts/minute, and always less than 20 acts/minute. |
| 18) As far as static EVLP is concerned, it is recommended to assess lung function after a recruitment manoeuvre aimed at reopening collapsed lung regions in order to prevent alveolar damage secondary to alveolar hypoxia and loss of surfactant and to counteract shunt. |
| 19) When performed for the purpose of static EVLP, it is preferable to perform the recruitment manoeuvres prior to graft insertion in the machine. |
| 20) When performed during EVLP, recruitment manoeuvres based on the application of tidal volume and/or high inspiratory pressures are not recommended. Transient increases in PEEP level are preferable. |
| 21) When safely performed, pronation of the lungs during EVLP is advisable. |
| 22) In case of 'minor' air leakage from the lung parenchyma that does not complicate parenchymal recruitment and organ assessment, attempting to repair parenchymal breaches with sutures or staplers is not recommended. |
| 23) In the event of dereclosure of the lung parenchyma or failure to achieve adequate recruitment in the absence of problems with the ventilation system, after verifying that the circuit has been properly closed and that there are no frank areas of parenchymal air leakage, flexible bronchoscopy through the dedicated operating channel is recommended to check for the presence of secretions and their possible aspiration. |
| 24) In the case of lung splitting during machine reperfusion, or in the case of monopulmonary reperfusion, the reduction of the tidal volume with respect to the criteria defined for bi-lung reperfusion is crucial. |
| 25) Once the suitability of the organs has been established, the lungs can be harvested and cooled at the same time or separated in the machine (cooling of the first lung to be implanted, maintenance of machine perfusion of the second lung). As there is currently no evidence to support a better quality of organs in the case of simultaneous removal of the lungs from the perfusion machine, or their separation (cooling of the first lung and preservation of the second by reperfusion), the decision as to the best time to separate the lungs (back table vs. EVLP system) remains at the discretion of the centre according to its preferences and availability. |
| 26) During reperfusion, the use of steroid therapy is recommended in order to reduce the risk of oedema. |
| 27) As there is no evidence at the present time to support a better result with the use of one class of antibiotics than the others, the decision on the use of the type of antibiotics during reperfusion is left to the experience of the transplant centre. |

**Table S3 - Initial statements – graft assessment parameters in EVLP**

| 1) The evaluation of graft quality on EVLP is based on multiple standard physiological and objective parameters. One parameter alone is not sufficient to assess graft quality. |
| --- |
| 2) For all evaluation parameters, the trend over time is more relevant than the absolute value (best or worst). |
| 3) A flexible bronchoscopy through a dedicated Bronco-Port is recommended to assess the presence of foamy fluid (signs of oedema), haemorrhagic fluid, repletion with purulent secretions or signs of aspiration. |
| 4) At least two endobronchial assessments during ex vivo perfusion are desirable. |
| 5) The use of grafts in which frank plasmorrhoea and signs of aspiration, or the repletion of purulent secretions are verified by bronchoscopy during EVLP, is not recommended. |
| 6) It is advisable, at the end of lung parenchyma recruitment, to visually inspect for features such as haemorrhagic infarction, the appearance of infarct areas, and other abnormalities that may affect lung function and its suitability for transplantation. |
| 7) After lung parenchyma recruitment, palpatory inspection of the graft is recommended to detect features such as reduced elasticity of the parenchyma itself or increased weight of the various areas, appearance of areas of thickening, and other abnormalities that may affect lung function and its suitability for transplantation. |
| 8) The evaluation of the PaO2/FiO2 value in isolation is never sufficient for the definitive assessment of the goodness of the graft. |
| 9) The evaluation of the graft and the PaO2/FiO2 value obtained in EVLP cannot be separated from the evaluation of the haemoglobin content in the perfusate. |
| 10) It is recommended, at the end of lung parenchyma recruitment and the performance of haemogasanalysis, to consider the difference in oxygen content between the arterial and venous side rather than the PaO2/FiO2 value. |
| 11) The use of grafts in which a PaO2/FiO2 value < 350mmHg is verified is not recommended after an adequate recruitment period and the performance of haemogasanalysis in EVLP. In spite of this, the assessment of graft quality is left to the experience of the transplant centre, depending on all the multiple physiological and objective parameters required for this assessment. |
| 12) In the event of a discrepancy between the haemogasanalysis and the other assessment parameters, the assessment of the selective contribution of the individual lung lobes is recommended. |
| 13) The evaluation of pulmonary vascular resistance trends during the procedure is recommended. An increase in resistance should be considered as organ damage. |
| 14) Continuous evaluation of perfusate leakage in the bell is recommended. Once anastomotic defects or frank parenchymal lesions have been excluded, evolution to pulmonary oedema should be considered. Where feasible, evaluation of weight change during the procedure may be an indication of possible organ oedema. |
| 15) Evaluation of static compliance of the isolated organ is recommended. Values below 70ml/cmH2O or worsening over time should be considered as graft damage. |
| 16) An X-ray is recommended, if possible, to better define any regionality of the organ damage (signs of oedema, imbibition, interstitial overload, parenchymal lesions); the X-ray alone cannot determine the use of the organ but only guide the decision. |

**Table S4 - Initial statements rewording – indications to EVLP**

| 1) EVLP can be used as an effective technique for organ preservation  [the concept of 'safety' of the procedure was removed, retaining instead that of 'effectiveness' in organ preservation] |
| --- |
| 2) EVLP is a useful platform for organ assessment |
| 3) There is currently no strong clinical evidence for a role of EVLP in active organ reconditioning  [it has been decided to further emphasise the absence, at the present time, of scientific evidence that absolutely justifies the use of EVLP machines for lung graft reconditioning] |
| 4) The use of EVLP may find indication in both DBD and DCD donation of any class |
| 5) EVLP can be used for graft, regardless of the clinical condition of the recipient |
| 6) The use of EVLP is proposed in cases of donors with questionable organ function, or not evaluable at harvest |
| 7) EVLP is usable in the case of logistical or clinical issues that have the potential to increase ischemia time |
| 8) EVLP is not recommended for use in case of irreversible structural damage of the graft |
| * Statement 9 was removed, as it was considered redundant with statement 1) of methods of use of EVLP |

**Table S5 - Initial statements rewording – methods of use**

| 1) There are three most widely used of EVLP in clinical practice (Lund, Toronto, OCS), but no evidence exists, at present, regarding the superiority of one over the others |
| --- |
| 2) The use of human albumin in the perfusate is recommended to improve the hyper-oncotic power of the perfusion solution.  [new wording: The use of human albumin in the perfusate is recommended to improve the hyper-oncotic power of the perfusion solution, although there is currently no strong evidence in the literature to support this] |
| 3) The addition of dextran to the solution limits the need for replacement of the hyper-oncotic solution over time.  [removed] |
| 4) Perfusion with solution supplemented with whole blood offers the best results in the preservation and homeostasis of the lung over time.  [removed] |
| 5) Perfusion with solution supplemented with whole blood has no application in the clinical setting due to the difficulties in obtaining it.  [removed] |
| 6) There are currently no differences in the clinical results obtained between perfusion with acellular solution and cellular solution with concentrated haematias. |
| 7) The achievement of the target flow must take place in a congruent time and concomitant with the heating of the lung. |
| 8) It is recommended to maintain systolic pulmonary arterial pressures below 20mmHg to reduce the risk of developing pulmonary oedema.  [new wording: It is recommended to maintain pulmonary arterial pressures less than 15-20mmHg to reduce the risk of developing pulmonary oedema] |
| 9) There are currently no differences in lung preservation results between the different target flows used with the different device protocols.  [now included in statement 1] |
| 10) In the case of lung splitting during in-machine reperfusion, as well as in monopulmonary reperfusion, adaptation of the target flow to the monopulmonary condition is mandated, while maintaining control of mean PAP and lung resistance as much as possible. |
| 11) There is no difference in pulmonary preservation results between closed and open atrium systems.  [now included in statement 1] |
| 12) Once the preferred atrium system is chosen, it is mandated that the atrial chamber is fully open or closed throughout the duration of perfusion.  [now included in statement 1] |
| 13) The goal of ventilation during reperfusion is to maintain protective ventilation settings (maintenance of low PEEP [5-7 cmH2O] and low tidal volume [approximately 5-7 mL/kg predicted donor body weight]) during functional evaluation of the organ. |
| 14) It is recommended that lung ventilation does not start until temperatures between 32-34°C have been reached. |
| 15) During the reperfusion process it is recommended to maintain low PEEP (5-7 cmH2O), even in the case of lungs that were initially de-recorded and visibly atelectatic at the time of donor retrieval.  [now included in statement 13] |
| 16) During the reperfusion process, the maintenance of low tidal volume (approximately 5-7 mL/kg predicted donor body weight) is recommended, even in the case of initially de-recorded and visibly atelectatic lungs at the time of donor collection.  [now included in statement 13] |
| 17) During the reperfusion process, it is recommended to maintain a respiratory rate of 7-12 acts/minute, and always less than 20 acts/minute. |
| 18) As far as static EVLP is concerned, it is recommended to assess lung function after a recruitment manoeuvre aimed at reopening collapsed lung regions in order to prevent alveolar damage secondary to alveolar hypoxia and loss of surfactant and to counteract shunt.  [new wording: Regarding static EVLP, it is recommended to assess lung function after a recruitment manoeuvre having the purpose of reopening collapsed lung regions] |
| 19) When performed for the purpose of static EVLP, it is preferable to perform the recruitment manoeuvres prior to graft insertion in the machine.  [new wording: When carried out for the purpose of static EVLP (delayed normothermic perfusion), it is preferable to perform hyperinflation manoeuvres to allow hypothermic transport prior to graft insertion.  or  When performed for the purpose of portable EVLP, it is not necessary to perform the hyperinflation manoeuvres as there is no hypothermic transport prior to graft insertion into the machine.] |
| 20) When performed during EVLP, recruitment manoeuvres based on the application of tidal volume and/or high inspiratory pressures are not recommended. Transient increases in PEEP level are preferable. |
| 21) When safely performed, pronation of the lungs during EVLP is advisable.  [new wording: Pronation of the lungs during EVLP can be considered] |
| 22) In case of 'minor' air leakage from the lung parenchyma that does not complicate parenchymal recruitment and organ assessment, attempting to repair parenchymal breaches with sutures or staplers is not recommended. |
| 23) In the event of dereclosure of the lung parenchyma or failure to achieve adequate recruitment in the absence of problems with the ventilation system, after verifying that the circuit has been properly closed and that there are no frank areas of parenchymal air leakage, flexible bronchoscopy through the dedicated operating channel is recommended to check for the presence of secretions and their possible aspiration. |
| 24) In the case of lung splitting during machine reperfusion, or in the case of monopulmonary reperfusion, the reduction of the tidal volume with respect to the criteria defined for bi-lung reperfusion is crucial. |
| 25) Once the suitability of the organs has been established, the lungs can be harvested and cooled at the same time or separated in the machine (cooling of the first lung to be implanted, maintenance of machine perfusion of the second lung). As there is currently no evidence to support a better quality of organs in the case of simultaneous removal of the lungs from the perfusion machine, or their separation (cooling of the first lung and preservation of the second by reperfusion), the decision as to the best time to separate the lungs (back table vs. EVLP system) remains at the discretion of the centre according to its preferences and availability.  [new wording: Once the suitability of organs has been established, as there is not enough literature at present, the decision of the best time of lung separation (back table vs. EVLP system) remains at the discretion of the centre according to its preferences, expertise and availability] |
| 26) During reperfusion, the use of steroid therapy is recommended in order to reduce the risk of oedema. |
| 27) As there is no evidence at the present time to support a better result with the use of one class of antibiotics than the others, the decision on the use of the type of antibiotics during reperfusion is left to the experience of the transplant centre.  [Since there is, at present, no evidence to support a better outcome with the use of one class of antimicrobials than the others, the decision on the use of the type and dosage of antimicrobials during EVLP is deferred to the experience of the transplant centre] |

**Table S6 - Initial statements rewording – graft assessment parameters in EVLP**

| 1) The evaluation of graft quality on EVLP is based on multiple standard physiological and objective parameters. One parameter alone is not sufficient to assess graft quality. |
| --- |
| 2) For all evaluation parameters, the trend over time is more relevant than the absolute value (best or worst). |
| 3) A flexible bronchoscopy through a dedicated Bronco-Port is recommended to assess the presence of foamy fluid (signs of oedema), haemorrhagic fluid, repletion with purulent secretions or signs of aspiration. |
| 4) At least two endobronchial assessments during ex vivo perfusion are desirable. |
| 5) The use of grafts in which frank plasmorrhoea and signs of aspiration, or the repletion of purulent secretions are verified by bronchoscopy during EVLP, is not recommended. |
| 6) It is advisable, at the end of lung parenchyma recruitment, to visually inspect for features such as haemorrhagic infarction, the appearance of infarct areas, and other abnormalities that may affect lung function and its suitability for transplantation. |
| 7) After lung parenchyma recruitment, palpatory inspection of the graft is recommended to detect features such as reduced elasticity of the parenchyma itself or increased weight of the various areas, appearance of areas of thickening, and other abnormalities that may affect lung function and its suitability for transplantation. |
| 8) The evaluation of the PaO2/FiO2 value in isolation is never sufficient for the definitive assessment of the goodness of the graft. |
| 9) The evaluation of the graft and the PaO2/FiO2 value obtained in EVLP cannot be separated from the evaluation of the haemoglobin content in the perfusate.  [Now included in statement 11] |
| 10) It is recommended, at the end of lung parenchyma recruitment and the performance of haemogasanalysis, to consider the difference in oxygen content between the arterial and venous side rather than the PaO2/FiO2 value.  [Now included in statement 11] |
| 11) The use of grafts in which a PaO2/FiO2 value < 350mmHg is verified is not recommended after an adequate recruitment period and the performance of haemogasanalysis in EVLP. In spite of this, the assessment of graft quality is left to the experience of the transplant centre, depending on all the multiple physiological and objective parameters required for this assessment.  [new wording: At the end of an adequate recruitment period and the performance of haemogasanalysis in EVLP, PaO2/FiO2 values <350mmHg (<300mmHg if cellular solution) indicate doubtful graft performance. In spite of this, the assessment of graft quality is left to the experience of the transplant centre, depending on all the many physiological and objective parameters required for this assessment.] |
| 12) In the event of a discrepancy between the haemogasanalysis and the other assessment parameters, the assessment of the selective contribution of the individual lung lobes is recommended.  [Now included in statement 11] |
| 13) The evaluation of pulmonary vascular resistance trends during the procedure is recommended. An increase in resistance should be considered as organ damage. |
| 14) Continuous evaluation of perfusate leakage in the bell is recommended. Once anastomotic defects or frank parenchymal lesions have been excluded, evolution to pulmonary oedema should be considered. Where feasible, evaluation of weight change during the procedure may be an indication of possible organ oedema. |
| 15) Evaluation of static compliance of the isolated organ is recommended. Values below 70ml/cmH2O or worsening over time should be considered as graft damage. |
| 16) An X-ray is recommended, if possible, to better define any regionality of the organ damage (signs of oedema, imbibition, interstitial overload, parenchymal lesions); the X-ray alone cannot determine the use of the organ but only guide the decision. |
